# Anti-GPR56 monoclonal antibody potentiates GPR56-mediated Src-Fak signaling to modulate cell adhesion

**DOI:** 10.1016/j.jbc.2021.100261

**Published:** 2021-01-08

**Authors:** Treena Chatterjee, Sheng Zhang, Tressie A. Posey, Joan Jacob, Ling Wu, Wangsheng Yu, Liezl E. Francisco, Qingyun J. Liu, Kendra S. Carmon

**Affiliations:** The Brown Foundation Institute of Molecular Medicine and Center for Translational Cancer Research, University of Texas Health Science Center at Houston, Houston, Texas, USA

**Keywords:** GPR56, ADGRG1, G-protein-coupled receptor (GPCR), monoclonal antibody, Src, focal adhesion kinase (Fak), paxillin, cell adhesion, colorectal cancer (CRC), 7TM, seven-transmembrane, ADGRG1, adhesion G protein-coupled receptor G1, aGPCR, adhesion G-protein-coupled receptor, CRC, colorectal cancer, CTF, C-terminal fragment, ECD, extracellular domain, Fak, focal adhesion kinase, GAIN, GPCR-autoproteolysis inducing, GPR56, G-protein-coupled receptor 56, GPS, GPCR Proteolysis Site, ICC, immunocytochemistry, KD, knockdown, mAb, monoclonal antibody, NTF, N-terminal fragment, Pax, paxillin, PLL, Pentraxin/Laminin/neurexin/sex-hormone(LNS)-binding-globulin-Like, RhoA, Ras homolog family member A, shRNA, short hairpin ribonucleic acid, siRNA, small interfering ribonucleic acid, Src, Src proto-oncogene, nonreceptor tyrosine kinase, SRE, serum response element, SRF-RE, serum response factor response element, STP, Serine–Threonine–Proline-rich, WT, wild-type

## Abstract

GPR56 is a member of the adhesion G-protein-coupled receptor family shown to play important roles in cell adhesion, brain development, immune function, and tumorigenesis. GPR56 is highly upregulated in colorectal cancer and correlates with poor prognosis. Several studies have shown GPR56 couples to the Gα_12/13_ class of heterotrimeric G-proteins to promote RhoA activation. However, due to its structural complexity and lack of a high-affinity receptor-specific ligand, the complete GPR56 signaling mechanism remains largely unknown. To delineate the activation mechanism and intracellular signaling functions of GPR56, we generated a monoclonal antibody (mAb) that binds with high affinity and specificity to the extracellular domain (ECD). Using deletion mutants, we mapped the mAb binding site to the GAIN domain, which mediates membrane-proximal autoproteolytic cleavage of the ECD. We showed that GPR56 overexpression in 293T cells leads to increased phosphorylation of Src, Fak, and paxillin adhesion proteins and activation of the Gα_12/13_-RhoA-mediated serum response factor (SRF) pathway. Treatment with the mAb potentiated Src-Fak phosphorylation, RhoA–SRF signaling, and cell adhesion. Consistently, GPR56 knockdown in colorectal cancer cells decreased Src–Fak pathway phosphorylation and cell adhesion. Interestingly, GPR56-mediated activation of Src–Fak phosphorylation occurred independent of RhoA, yet mAb-induced potentiation of RhoA–SRF signaling was Src-dependent. Furthermore, we show that the C-terminal portion of the Serine–Threonine–Proline-rich (STP) region, adjacent to the GAIN domain, was required for Src–Fak activation. However, autoproteolytic cleavage of the ECD was dispensable. These data support a new ECD-dependent mechanism by which GPR56 functions to regulate adhesion through activation of Src–Fak signaling.

GPR56, also referred to as *ADGRG1*, is a member of the adhesion G-protein-coupled receptor (aGPCR) family with a large N-terminal extracellular domain (ECD) that is thought to function in mediating cell–cell and cell–matrix interactions (1). GPR56 has been shown to be critical for many physiological processes, including immune function (2), maintenance of hematopoietic stem cells (3), and oligodendrocyte and cortex development (4, 5, 6, 7). Mutations in GPR56 have been associated with bilateral frontoparietal polymicogyria (BFPP), a disorder characterized by a disruption in the organization of the frontal cortex (6, 8). Recently, GPR56 has been implicated in contributing to tumorigenesis of various types of cancer. GPR56 is upregulated in cancers of the breast, lung, ovary, pancreas, colon, and glioblastoma (9, 10, 11). Analysis of clinical data revealed a significant correlation between high *GPR56* levels and poor outcome in acute myeloid leukemia, ovarian cancer, and colorectal cancer (CRC) (10, 11, 12, 13, 14, 15). In CRC, GPR56 was shown to promote drug resistance and drive tumor growth (12, 13, 16). On the contrary, GPR56 has been shown to be downregulated in metastatic melanoma and inhibitory to melanoma growth and metastasis (17). These studies demonstrate the essential functions of GPR56 and its emerging, yet diverse roles in tumor progression.

Similar to other aGPCRs, the ECD of GPR56 is structurally characterized by the presence of a highly conserved GPCR-Autoproteolysis INducing (GAIN) domain featuring a juxtamembrane GPCR Proteolysis Site (GPS) (1, 18). The GPS can be autoproteolytically cleaved, leaving two noncovalently associated but distinct fragments: (1) the N-terminal fragment (NTF), which consists of a Pentraxin and Laminin/neurexin/sex-hormone(LNS)-binding-globulin-Like (PLL) domain with adhesion properties, an overlapping Serine–Threonine–Proline-rich (STP) region, and the bulk of the GAIN domain; (2) and the C-terminal fragment (CTF), which incorporates the C-terminal region of the GAIN domain, referred to as the stalk or *Stachel*, and the seven-transmembrane region (7TM) (1, 19). Typically, aGPCR activation involves binding of the NTF with its ligand followed by activation of the CTF to transmit intracellular signaling (1). Evidences supporting both stalk-dependent and stalk-independent models of ligand-induced GPR56 activation have been reported; however, the exact mechanism remains elusive. In the stalk-dependent model, the NTF serves as a shield for the stalk region and upon ligand binding the NTF dissociates or sheds from the CTF, allowing stalk to function as a “tethered agonist” that can engage with the 7TM (20, 21, 22). Alternatively, the stalk-independent model suggests that a ligand-induced conformational change of the ECD (incorporating the NTF and the stalk region), which promotes interaction with the 7TM, is required to stimulate signaling activity (20, 23). GPR56 has been shown to interact with proteins such as collagen III (24), transglutaminase 2 (TG2) (17), and progastrin (16); however, receptor-specific ligands have yet to be fully validated. Several studies have established that GPR56 is coupled to the Gα_12/13_ class of heterotrimeric G-proteins to promote RhoA activation (13, 22, 24, 25, 26). GPR56 overexpression has been shown to activate various signaling pathway response elements, including serum response element (SRE) and serum response factor response element (SRF-RE) that are likely regulated downstream of RhoA (20, 21, 25). However, due to its structural complexity and lack of high-affinity receptor-specific ligands, the activation and intracellular signaling mechanism(s) of GPR56 have yet to be completely resolved.

In this study, we generated a high-affinity anti-GPR56 monoclonal antibody (mAb) 10C7, which binds to the GAIN domain, to investigate the activation and signaling mechanisms of GPR56. We show that GPR56 can activate Src–Fak adhesion signaling in 293T and CRC cells. GPR56-mediated activation of this pathway was independent of autoproteolytic cleavage of the ECD and required the STP region, indicating the importance of the NTF. Treatment with 10C7 potentiated both RhoA–SRF and Src–Fak signaling. GPR56-mediated activation of Src–Fak occurred independent of RhoA. However, activation of Src was required for 10C7-induced potentiation of RhoA–SRF signaling. Overall, we demonstrate a new ECD-dependent mechanism by which GPR56 functions to regulate cell adhesion through activation of the Src–Fak pathway.

## Results

### Characterization of monoclonal antibody 10C7 targeted to the ECD of GPR56

**Figure 1:**
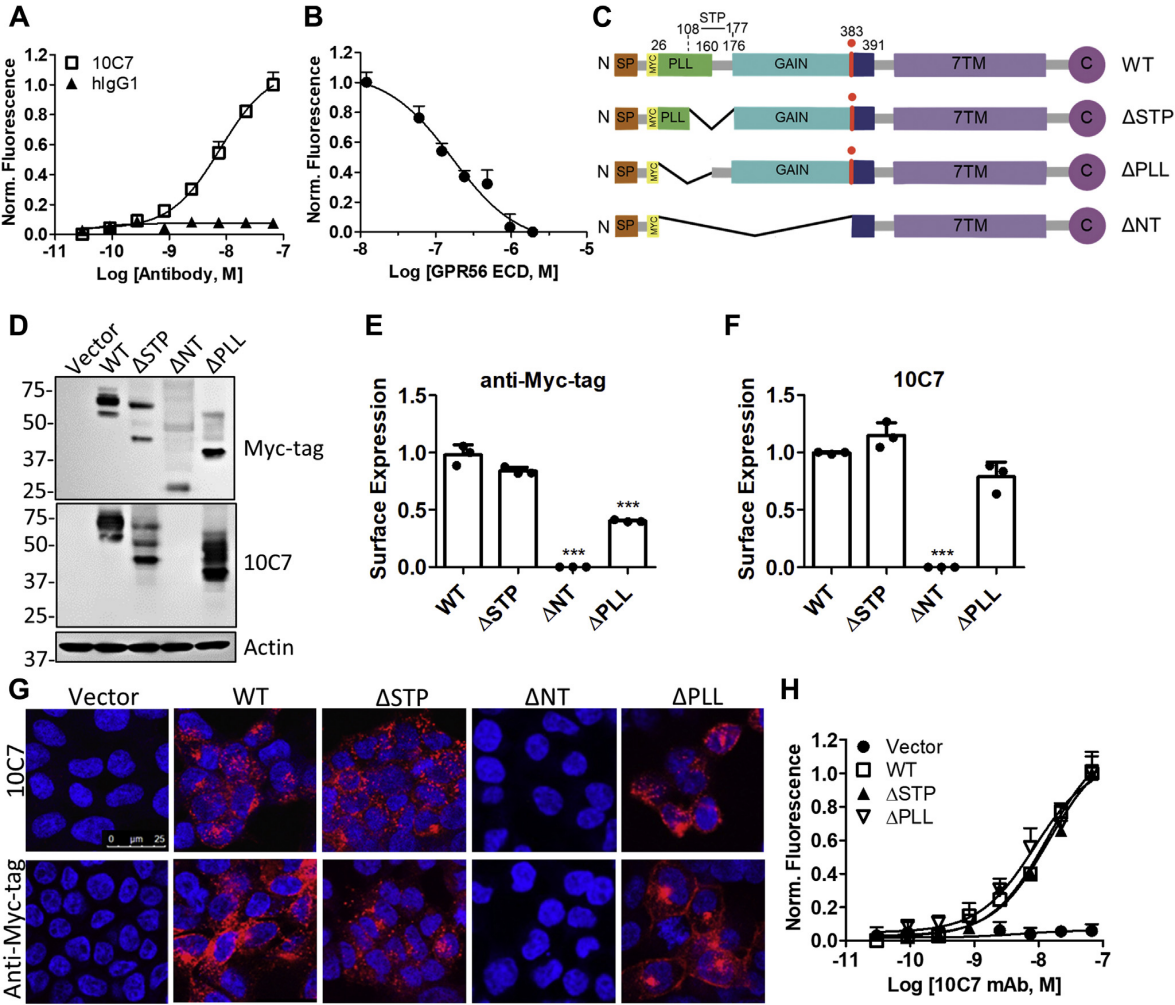
**Novel Anti-GPR56 mAb 10C7 binds the GAIN domain of GPR56 with high specificity.***A*, cell-based binding assay shows 10C7, but not hIgG1(isotype control) binds 293T-GPR56 WT cells and *B*, purified GPR56 ECD inhibits 10C7 binding to 293T-GPR56 WT cells. Error bars, S.E. *C*, schematic of the structure of GPR56 deletion mutants. *D*, western blot of vector, GPR56 WT, and deletion mutants in stable 293T cells using myc-tag and 10C7 mAbs. *E* and *F*, quantification of relative surface expression of receptors based on *E*, myc-tag mAb or *F*, 10C7 binding to live cells at 4 °C. Error bars, S.D. *G*, confocal microscopy images of 10C7 (5 μg/ml or 33 nM) and myc-tag mAb binding and internalization in stable cell lines after treatment for 1 h at 37 °C. *H*, cell-based binding assay shows 10C7 binds WT, ΔSTP, and ΔPLL, but not vector cells in a concentration-dependent manner. Error bars, S.E. All data represent at least three independent experiments.

We generated and purified a unique mouse–human chimera anti-GPR56 mAb 10C7 directed against the ECD of GPR56 (amino acids 1–400). To characterize 10C7, we first established HEK293T (293T) cell lines with stable overexpression of myc-tagged full-length human GPR56 wild-type (293T-GPR56 WT). To determine relative binding affinities, we employed a cell-based fluorescence binding assay. We showed that 10C7 binds GPR56 with high affinity and approximate K_d_ of 1.2 μg/ml or 7.8 nM (Fig. 1*A*). No binding was detected for human IgG1 isotype control. To further demonstrate specificity, a competitive binding assay was performed with the purified GPR56 ECD used for immunization during mAb production. As shown in Figure 1*B*, preincubation of 5 μg/ml (or 33 nM) 10C7 with ECD inhibited 10C7 binding to GPR56 at the cell surface in a concentration-dependent manner. Confocal analysis further confirmed 10C7 binding was blocked by ECD in 293T-GPR56 WT cells (Fig. S1*A*). To map the domain of the ECD to which 10C7 binds, we generated a series of truncation and deletion mutants and established stable overexpression cell lines (Fig. 1*C*). These cell lines include mutants of full-length GPR56 lacking the STP region (a.a.108–177; ΔSTP), deletion of the PLL domain (a.a. 26–160; ΔPLL), deletion of the NTF (a.a. 26–383; ΔNT) or vector control. The STP region encompasses the C-terminal portion of the PLL domain, the PLL–GAIN linker (a.a.161–175) and first two amino acids of the GAIN domain (19, 27). Previously, it has been shown that the STP region is required for GPR56 interaction with TG2 (27), and the PLL domain has been shown to bind collagen III (24), suggesting these regions may play an important role in ligand/protein interactions. Stable overexpression of GPR56 was verified by western blot using myc-tag antibody and 10C7 (Fig. 1*D*). Of note, the three major molecular weight bands for GPR56 represent expression of glycosylated full-length (top), partially, or unglycosylated full-length (middle) and cleaved ECD (bottom), as has been previously reported (21). Interestingly, 10C7 failed to detect expression of ΔNT, suggesting that under denaturing conditions 10C7 does not bind the stalk region of GPR56. Given that 10C7 binds to both native and denatured forms of GPR56 indicates that the mAb binds a surface exposed, linear epitope. To further verify the region where the 10C7 epitope resides, we performed cell-based fluorescence assays at 4ºC and quantified relative surface expression of GPR56 and its mutants in live cells using 5 μg/ml (or 33 nM) 10C7 or myc-tag mAbs (Fig. 1, *E* and *F*). Immunocytochemistry (ICC) and confocal analysis was performed to visualize binding and mAb-receptor cointernalization after mAb treatment of live cells for 1 h at 37 °C (Fig. 1*G*). Our findings showed that similar levels of GPR56 WT and ΔSTP were detected at the cell surface based on 10C7 and myc-tag mAb binding, whereas the ΔPLL mutant showed reduced surface expression (Fig. 1, *E* and *F*). Furthermore, GPR56 WT, ΔSTP, and ΔPLL cointernalized with both mAbs (Fig. 1*G*). No surface binding nor internalization was detected for either 10C7 or myc-tag mAb in vector or ΔNT cells (Fig. 1, *E*–*G*). However, ICC of fixed, permeabilized ΔNT cells showed binding of myc-tag mAb and not 10C7, as was demonstrated by western blot (Fig. S1*B* and Fig. 1*D*). While these findings suggest an impairment in the ΔNT mutant for trafficking to the cell surface, more importantly, they confirm that 10C7 does not bind the stalk region of GPR56. We then determined relative binding affinities of 10C7 for each of the GPR56 deletion mutants. 10C7 showed similar, high-affinity binding for WT, ΔSTP, and ΔPLL with average K_d_ of ∼7.8 nM, 8.1 nM, and 8.8 nM, respectively (Fig. 1, *A* and *H*). No binding of 10C7 was detected in vector cells (Fig. 1*H*). These findings indicate that 10C7 binds GPR56 ECD within the GAIN domain of the NTF with high affinity and specificity and does not bind the CTF stalk region.

### GPR56-mediated basal SRF-RE activity is dependent on the N-terminal of the PLL domain

**Figure 2:**
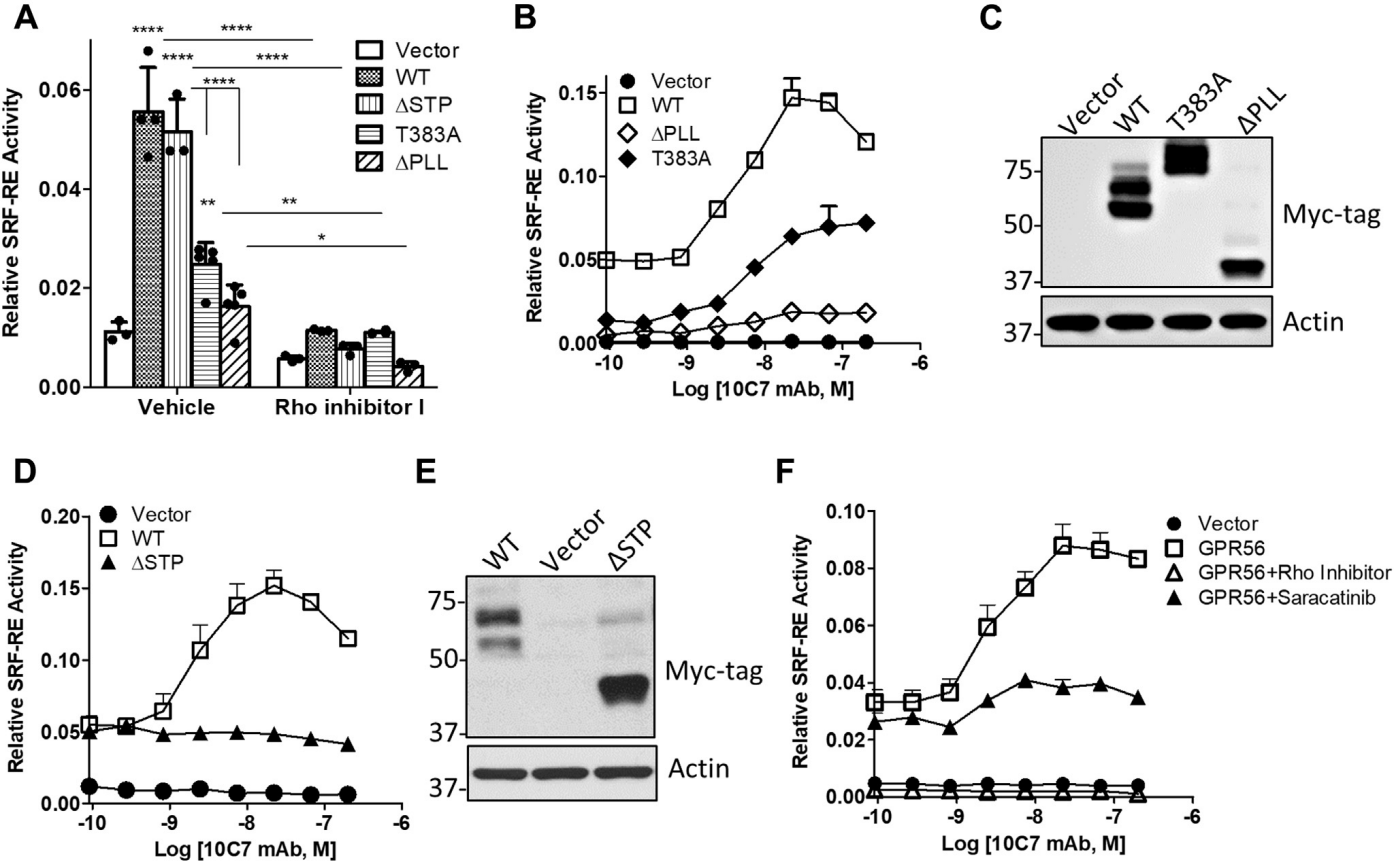
**10C7 potentiates GPR56 activation of RhoA-mediated SRF signaling independent of receptor cleavage, but requires the STP domain.***A*, transient overexpression of GPR56 WT, ΔSTP, and T383A in 293T cells significantly enhances SRF-RE activity, which is blocked by pretreatment with Rho inhibitor I (2 μg/ml) for 3 h. Statistical significance determined by ANOVA, ∗∗∗∗*p* < 0.0001. Error bars, S.D. *B*, 10C7 induces a dose-dependent potentiation of SRF-RE in WT, T383A, and ΔPLL transfected cells. Error bars, S.E. *C*, western blot of GPR56 WT, T383A, and ΔPLL overexpression. *D*, 10C7 does not potentiate in ΔSTP transfected cells. Error bars, S.E. *E*, western blot of GPR56 WT and ΔSTP overexpression. *F*, effects of Rho inhibitor I and Src inhibitor (saracatinib) on 10C7 potentiation of GPR56-meditated SRF signaling. Error bars, S.E. All data represent at least three independent experiments.

GPR56 has been shown to induce activation of the small GTPase RhoA mediated through Gα_12/13_ to promote cell adhesion (13, 20, 24, 25, 26). To examine if GPR56 deletion mutants were fully functional and dependent on RhoA activity, we employed the SRF-RE reporter assay, which drives transcription of a luciferase reporter gene in response to G_12/13_-RhoA-mediated activation of SRF signaling. SRF binding to its response element promotes transcription of target genes involved in the regulation of cell adhesion and actin dynamics (28). A series of reports have shown that GPR56 can activate both SRF-RE (20) and the SRE reporter (21, 23, 29). SRF-RE is similar to SRE, yet is a more precise readout for G_12/13_-RhoA-mediated signaling (30). Transient overexpression of GPR56 WT or ΔSTP significantly increased SRF-RE activity (approximately fivefold) compared with vector (Fig. 2*A*). Contrastingly, ΔPLL did not significantly activate SRF-RE. To test if NTF dissociation was required for signaling, we generated the GPS cleavage-deficient mutant, T383A, as previously reported (20). We found that this mutant routinely exhibited a two- to threefold reduction in SRF-RE activation compared with WT. Of note, T383A exhibited similar surface expression as WT and also cointernalized with 10C7 (Fig. S1, *C* and *D*). Treatment with a Rho inhibitor suppressed SRF-RE response and active RhoA levels (Fig. 2*A* and Fig. S1*E*), confirming that GPR56-mediated SRF-RE activity is mediated through RhoA. These data suggest that the PLL domain (a.a. 26–108, exclusive of the STP) is important for basal constitutive GPR56-mediated RhoA–SRF signaling, yet the STP region is dispensable. Furthermore, these findings suggest that although GPS-mediated autoproteolytic cleavage of the ECD is not required for RhoA–SRF signaling, it may be essential for maximal basal activity.

### 10C7 potentiates SRF-RE activity independent of ECD autoproteolysis, but requires the C-terminal of the STP region and activation of Src

Previously, we and others have demonstrated that mAbs targeting 7TM receptors can function to activate intracellular signaling (31, 32, 33). Therefore, we tested whether 10C7 could modulate GPR56-mediated SRF-RE activity. 293T cells were cotransfected with the vector control or GPR56 expression plasmids and SRF-RE then treated with 10C7 in a dose-dependent manner. Interestingly, we found that 10C7 induced SRF-RE activity by an average of threefold in GPR56 WT, T383A, and ΔPLL with EC_50_s of ∼2 nM, 8 nM, and 5 nM, respectively (Fig. 2*B*). GPR56 expression was confirmed by western blot (Fig. 2*C*). No 10C7-induced effects were observed in ΔSTP or vector transfected cells (Fig. 2, *D* and *E*). These findings suggest that 10C7 activates GPR56-mediated SRF signaling in a STP-dependent fashion. Since the STP includes the C-terminal portion of the PLL, this suggests that the region of the STP spanning the PLL-GAIN linker (a.a. 161–177) may be required for 10C7 potentiation SRF-RE activity.

To identify potential new molecular players contributing to 10C7-induced potentiation of GPR56-mediated RhoA–SRF activity, we screened a variety of chemical inhibitors utilizing the SRF-RE assay. As expected, treatment with Rho inhibitor completely suppressed constitutive and 10C7-induced SRF-RE response in GPR56 WT cells (Fig. 2*F*) and basal response in vector cells (Fig. S1*F*). Interestingly, we found that treatment with saracatinib, an inhibitor of the Src family of tyrosine kinases (Src), suppressed 10C7 potentiation of the SRF-RE reporter, yet only had minimal effect on baseline activity in GPR56 WT cells (Fig. 2*F*). Saracatinib has no effect on SRF-RE activity in vector cells (Fig. S1*F*). These results demonstrate that Src activation may play a role in 10C7-induced activation of GPR56-mediated SRF signaling.

### GPR56 activates Src–Fak adhesion signaling and is potentiated by 10C7

**Figure 3:**
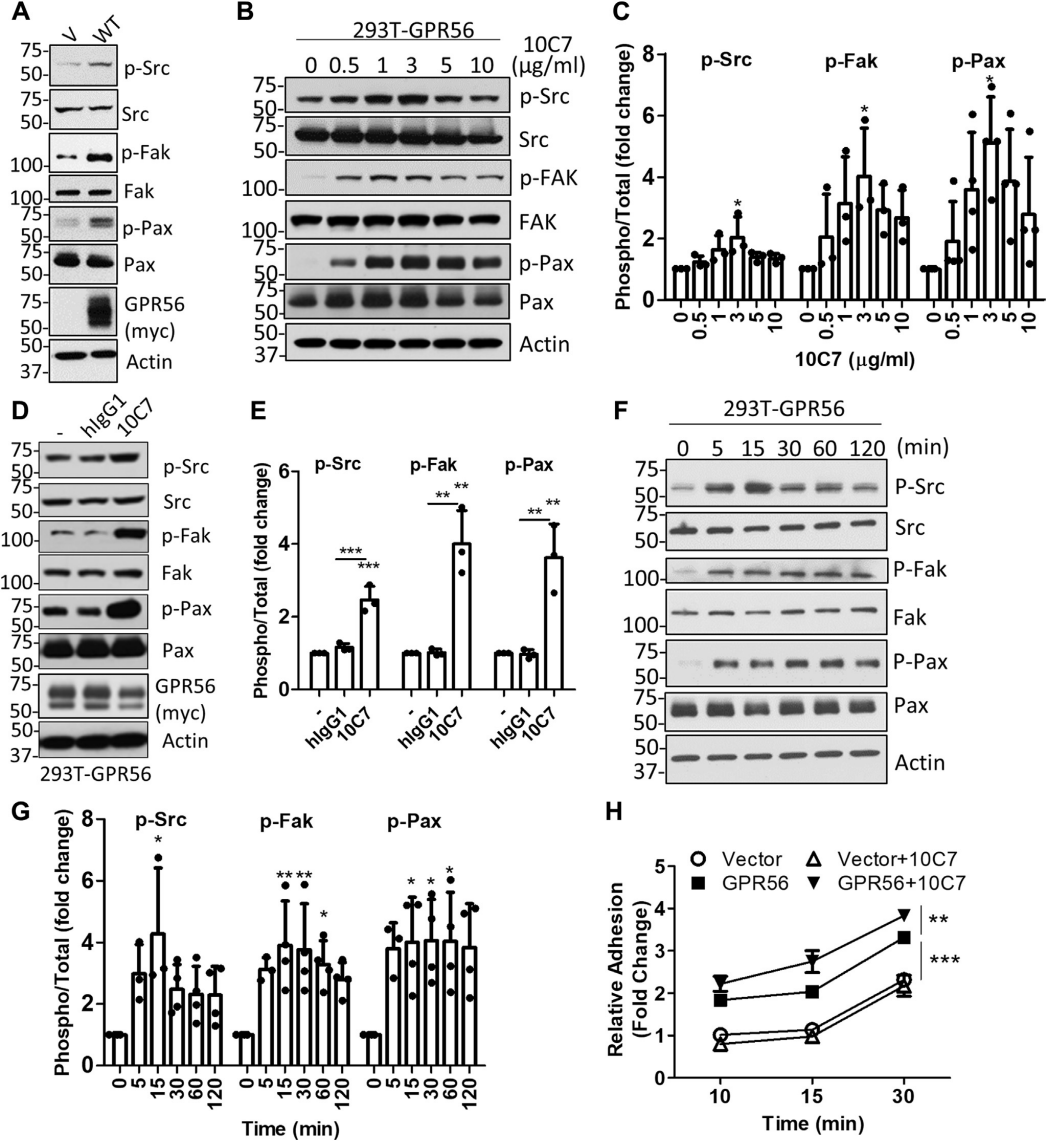
**GPR56 activates Src–Fak adhesion signaling in 293T cells.***A*, western blot showing transient transfection of GPR56 WT activates phosphorylation of Src, Fak, and paxillin. *B*, 10C7 dose-dependent phosphorylation in serum starved 293T-GPR56 stable cells treated for 1 h. *C*, quantification of 3 to 4 independent experiments as shown in *B*. *D*, 10C7 and not isotype control antibody (hIgG1) potentiates Src–Fak signaling in GPR56 transfected cells after 30 min. *E*, quantification of three independent experiments as shown in *D*. *F*, time-dependent effects on phosphorylation in serum starved 293T-GPR56 stable cells treated with 10C7 (3 μg/ml or 20 nM). *G*, quantification of 3 to 4 independent experiments as shown in *F*. *H*, 293T-GPR56 cells exhibit increased adhesion to collagen, which is augmented by 10C7. Data graphed as fold change *versus* vector cells at the 10 min time point. Statistical significance for adhesion assay determined by two-way ANOVA (Error bars, S.E.) and western blots by one-way ANOVA (Error bars, S.D.), ∗*p* < 0.05, ∗∗*p* < 0.01, and ∗∗∗*p* < 0.001.

Since Src and focal adhesion kinase (FAK) are widely associated with adhesion signaling, we tested if overexpression of GPR56 could modulate Src–Fak activity. As shown in Figure 3*A*, transient transfection of GPR56 showed increased phosphorylated levels of Src, Fak, and paxillin (Pax) compared with vector. This was also confirmed in GPR56 WT stable cells (Fig. S2*A*). Next, we tested if 10C7 treatment could potentiate phosphorylation. Dose-dependent treatment of GPR56 WT stable cells showed that maximum phosphorylation was induced by 3 μg/ml (or 20 nM) 10C7 after 1 h (Fig. 3, *B* and *C*). No change in phosphorylation was detected after treatment of GPR56 WT cells with a nontargeting hIgG1 isotype control antibody (Fig. 3, *D* and *E*) or myc-tag mAb, which binds to the N terminus of recombinant GPR56 WT (Fig. S2*B*). 10C7 and myc-tag mAb were shown to similarly cointernalize with GPR56 (Fig. 1*G* and Fig. S2*C*), suggesting that 10C7 potentiation of GPR56-mediated Src–Fak activity is a result of its binding to the GAIN domain and not changes in the extent of receptor internalization. Time-dependent treatment of GPR56 WT cells with 3 μg/ml (or 20 nM) showed increased phosphorylation of Src, Fak, and Pax at 5 min with peak phosphorylation of Src observed at 15 min posttreatment (Fig. 3, *F* and *G*). A longer time course showed that elevated phosphorylation levels were still detected after 6 h posttreatment with 10C7 in GPR56 WT stable cells, and no significant changes in phosphorylation were detected in vector stable cells (Fig. S2*A*). Notably, we repeatedly observed a second peak activation of Src phosphorylation, which may be attributed to recycling of 10C7 and restimulation of GPR56. Since GPR56 drives cell adhesion and has been shown to bind collagens (24, 34, 35), we investigated if pretreatment with 10C7 could enhance adhesion to a collagen in a GPR56-specific manner. Our results show that GPR56 expression increased adhesion of 293T cells compared with vector, with approximate twofold increase observed after 10 min (Fig. 3*H*). Furthermore, 10C7 treatment significantly increased the rate of adhesion of GPR56 WT cells after 15 min, yet had no effect on vector cells. These findings demonstrate that 10C7 can potentiate GPR56-mediated Src–Fak signaling and cell adhesion in 293T cells.

### GPR56-mediated Src–Fak signaling occurs independent of ECD cleavage and requires the STP region

**Figure 4:**
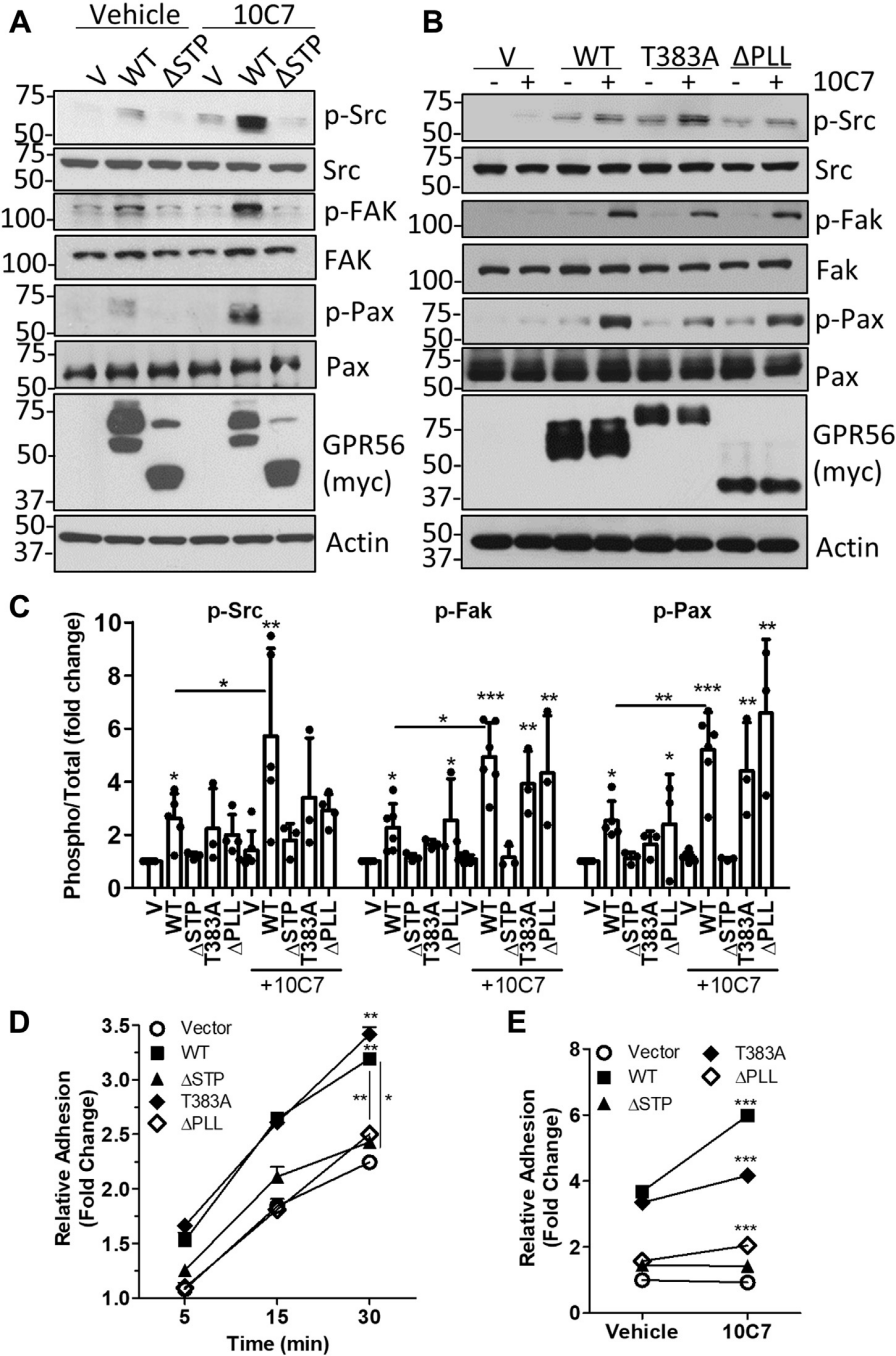
**GPR56 activates Src–Fak adhesion signaling in 293T cells and requires the STP domain.** Western blot showing effects of GPR56 WT compared with *A*, ΔSTP or *B*, T383A and ΔPLL mutants on phosphorylation of Src, Fak, and paxillin ± 10C7 (3 μg/ml or 20 nM) treatment for 1 h. *C*, quantification of 3 to 5 independent experiments as shown in *A* and *B*. *D*, time-dependent and *E*, 10C7-induced effects on adhesion of 293T GPR56 WT and mutant stable cell lines to collagen. Cell adhesion in ±10C7 treatment was quantified after 15 min. Statistical significance for adhesion assays determined by two- or one-way ANOVA (Error bars, S.E.) and western blots by one-way ANOVA (Error bars, S.D.), ∗*p* < 0.05, ∗∗*p* < 0.01, and ∗∗∗*p* < 0.001 compared with vector cells or untreated cells for overexpression and 10C7 treatment studies, respectively, unless otherwise indicated. All data represent at least three independent experiments.

Next, we tested if deletion of the PLL or STP regions of the ECD or cleavage-deficient point mutation T383A could alter GPR56-mediated Src–Fak signaling (Fig. 4, *A*–*C*). Western blot showed that unlike GPR56 WT, stable overexpression of ΔSTP had no effect on phosphorylation of Src, Fak, or Pax (Fig. 4, *A* and *C*). However, T383A and ΔPLL stable cell lines showed increased basal phosphorylation levels similar to WT cells. Treatment with 10C7 (3 μg/ml or 20 nM) considerably enhanced phosphorylation of Src, Fak, and Pax in WT, T383A, and ΔPLL cells, but not in ΔSTP cells (Fig. 4, *A*–*C*). These findings were also confirmed by transient overexpression (Fig. S2, *D* and *E*). We then investigated the effects of GPR56 mutant overexpression on adhesion to collagen. As shown in Figure 4*D*, the rate of T383A cell adhesion was significantly increased compared with vector cells and similar to that of WT. However, stable overexpression of ΔSTP or ΔPLL did not have a significant effect. 10C7 treatment significantly increased adhesion of WT, T383A, and ΔPLL cells after 15 min, yet had no effect on ΔSTP or vector cells. These results suggest that the STP region of GPR56 is required for Src–Fak signaling and adhesion; however, autoproteolytic cleavage of the ECD is not essential.

### Depletion of GPR56 decreases Src–Fak phosphorylation and adhesion of colorectal cancer cells

**Figure 5:**
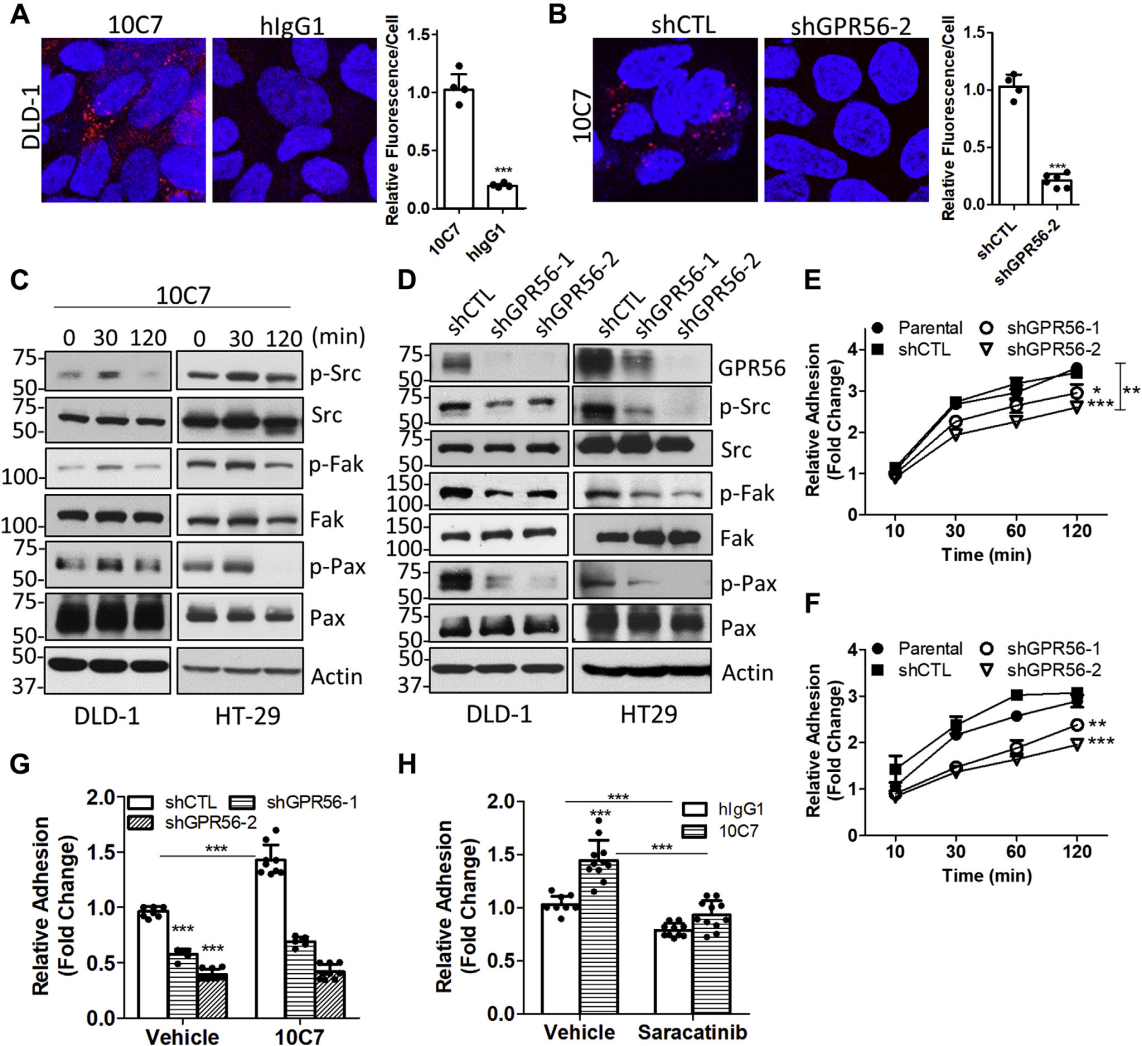
**GPR56 regulates Src–Fak phosphorylation and adhesion in colorectal cancer cells.***A*, confocal microscopy images and quantification of ICC showing 10C7 (15 μg/ml or 100 nM) binds GPR56 and internalizes in DLD-1 cells after 1 h at 37 °C. No binding was detected using nontargeting hIgG1 isotype control. *B*, 10C7 binds shRNA control (shCTL), but not GPR56 shRNA knockdown DLD-1 cells (shGPR56-2). Statistical significance determined by Student’s *t*-test, ∗∗∗*p* < 0.001 Error bars, S.D. *C*, western blot showing time-dependent effects of 10C7 (20 μg/ml or 130 nM) treatment on phosphorylation of Src, Fak, and paxillin in serum starved DLD-1 and HT-29 cells. *D*, GPR56 knockdown decreases phosphorylation of Src, Fak, paxillin and *E* and F, reduces collagen adhesion of E, DLD-1 and F, HT-29 cells. Statistical significance determined by two-way ANOVA. For DLD-1 cells, ∗*p* < 0.05, and ∗∗∗*p* < 0.001 compared with shCTL and ∗∗*p* < 0.01 for shGPR56-2 compared with parental cells. For HT-29 cells, ∗∗*p* < 0.01, and ∗∗∗*p* < 0.001 compared with shCTL and parental cells. Error bars, S.E. *G*, 10C7 (20 μg/ml or 130 nM) increases adhesion of HT-29 shCTL cells, but not GPR56 knockdown cells after 30 min. *H*, Src inhibitor, saracatinib (10 μM), decreases 10C7-induced adhesion in HT-29 cells after 30 min. Statistical significance for *G* and *H* determined by one-way ANOVA, ∗∗∗*p* < 0.001. Error bars, S.D. All data represent at least three independent experiments.

Similar to GPR56, Src and Fak are upregulated in CRC and high expression correlates with poor clinical prognosis (12, 13, 36, 37, 38, 39, 40). Therefore, we investigated if 10C7 could specifically bind endogenous GPR56 to activate Src–Fak signaling in CRC cell lines. Previously, we reported that DLD-1 and HT-29 cells express high levels of GPR56, and we established GPR56 shRNA knockdown (KD) cell lines (13). ICC and confocal analysis showed 10C7 binding and internalization in DLD-1 cells after 1 h at 37 °C (Fig. 5*A*). No binding of the hIgG1 isotype control was detected. Additionally, 10C7 did not bind DLD-1 GPR56 KD (shGPR56) cells, yet shRNA control (shCTL) cells showed similar binding and internalization as observed in parental DLD-1 cells (Fig. 5*B*). These results validate 10C7 binding specificity for endogenous GPR56. We then tested if treatment of CRC cells with 10C7 could activate Src–Fak signaling. Cells were serum starved and treated with 20 μg/ml (or 130 nM) 10C7 at the indicated time points (Fig. 5*C*). Interestingly, both cell lines showed increased levels of Src, Fak, and Pax phosphorylation at 30 min that returned to baseline levels or below at 2 h (Fig. 5*C* and Fig. S3*A*). To determine if ablation/depletion of endogenous GPR56 expression affects Src–Fak signaling, we performed western blot using our previously established shCTL and shGPR56 DLD-1 and HT-29 cell lines (13). GPR56 KD using two different shRNAs resulted in an approximate 85 to 90% reduction in GPR56 protein expression in DLD-1 cells and 60 to 90% in HT-29 cells (Fig. 5*D* and Fig. S3*B*). Loss of GPR56 decreased phosphorylated levels of Src, Fak, and Pax, indicating that endogenous GPR56 mediates Src–Fak signaling exclusive of 10C7 treatment (Fig. 5*D* and Fig. S3*C*). We then tested if GPR56 KD effected cell adhesion. As shown in Figure 5, *E* and *F*, loss of GPR56 expression in both DLD-1 and HT-29 cells significantly decreased the rate of cell adhesion to collagen matrix. Since, GPR56 KD in HT-29 cells showed a greater change in cell adhesion compared with DLD-1 cells, we utilized the HT-29 cell lines to test the effect of 10C7 on adhesion. As shown in Figure 5*G*, 10C7 treatment significantly increased adhesion of HT-29 shCTL cells after 30 min, yet had no significant effect on GPR56 KD cells. To investigate if the 10C7-induced increase in cell adhesion is mediated through Src, HT-29 cells were treated with 10C7 or hIgG1 isotype control antibody in the presence or absence of the Src inhibitor, saracatinib (10 μM). Figure 5*H* shows that inhibition of Src significantly suppressed 10C7-induced cell adhesion. These data demonstrate that GPR56 can regulate Src–Fak adhesion signaling in CRC cells.

### GPR56-mediated Src–Fak phosphorylation is independent of Gα_12/13_, Gα_q_, and RhoA activation

**Figure 6:**
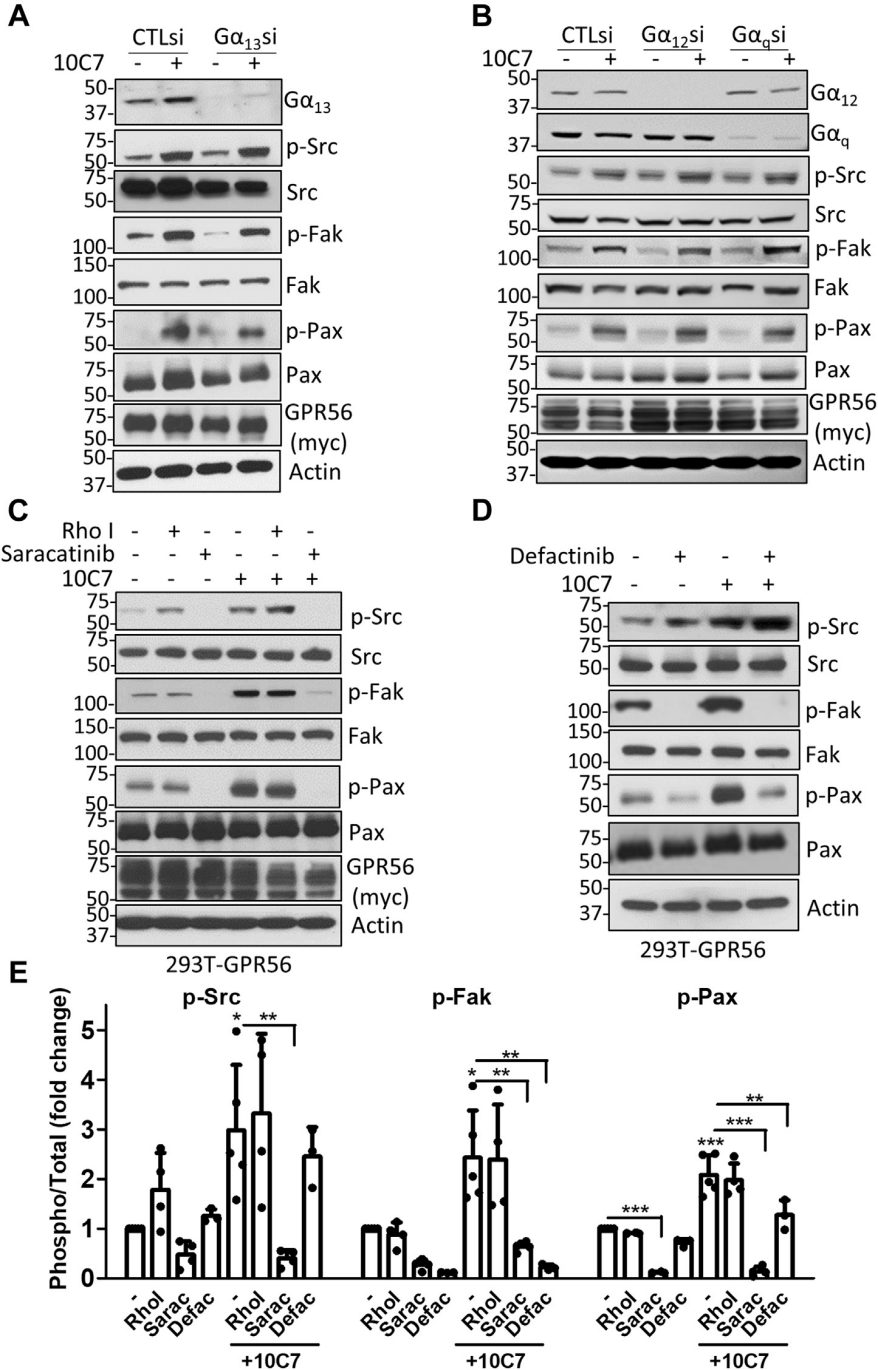
**GPR56-mediated phosphorylation of Src occurs upstream of Fak and is independent of RhoA activation.** Western blots showing knockdown of *A*, G⍺_13_ and *B*, G⍺_12_ or G⍺_q_ does not significantly affect GPR56-mediated Src/Fak signaling in 293T cells. *C* and *D*, western blots showing effect of inhibitors of *C*, RhoA (Rho Inhibitor I, 2 μg/ml), Src (saracatinib, 10 μM), and *D*, Fak (defactinib, 10 μM) on constitutive and 10C7-induced phosphorylation in 293T-GPR56 stable cells. Cells were pretreated with inhibitors for 3 h, then treated with 10C7 (3 μg/ml or 20 nM) for 45 min. *E*, quantification of at least three independent experiments as shown in *C* and *D*. Statistical significance for western blots was performed by one-way ANOVA, ∗*p* < 0.05, ∗∗*p* < 0.01, and ∗∗∗*p* < 0.001. Error bars, S.D.

Since we found that 10C7 potentiation of GPR56-mediated SRF-RE signaling required Src, we wanted to determine if phosphorylation of Src, Fak, and Pax was dependent on Gα_12/13_ and activation of RhoA. 293T cells were cotransfected with GPR56 and either Gα_12_-or Gα_13_-targeting or control siRNA and treated with or without 10C7. As shown in Figure 6, *A* and *B* and Fig. S4, *A* and *B*, knockdown of either Gα_12_ or Gα_13_ did not have a significant effect on baseline or 10C7-induced phosphorylation mediated by GPR56. Double knockdown of Gα_12_/Gα_13_ was also performed to confirm that there was no compensation between Gα_12_ and Gα_13_ activity (Fig. S4*C*). In addition to Gα_12/13_, Gα_q_ has also been shown to mediate RhoA signaling (41, 42). However, siRNA knockdown of Gα_q_ had no effect on GPR56-mediated Src–Fak pathway phosphorylation (Fig. 6*B* and Fig. S4, *A* and *B*). To verify that siRNA knockdown of Gα subunits was sufficient to impair downstream signaling, we showed that loss of Gα_13_ significantly inhibited GPR56-mediated SRF-RE reporter activity (Fig. S4*D*). We then examined if RhoA activation is required for GPR56-mediated phosphorylation of Src, Fak, and Pax. 293T-GPR56 cells were pretreated with Rho inhibitor for 3 h and then treated with or without 10C7. Results showed that treatment with Rho inhibitor failed to suppress phosphorylation (Fig. 6, *C* and *E*), suggesting that GPR56-mediated activation of RhoA is not essential for activation of Src–Fak signaling. In fact, pretreatment with the Rho inhibitor leads to a slight elevation in baseline p-Src levels. Next, we investigated whether Src or Fak phosphorylation occurs immediately following GPR56 activation. We pretreated 293T-GPR56 cells with either Src inhibitor (saracatinib) or Fak inhibitor (defactinib) for 3 h, then treated with or without 10C7 (Fig. 6, *C*–*E*). As shown in Figure 6, *C* and *E*, saracatinib inhibited both basal and 10C7-induced phosphorylation of Src, Fak, and Pax. Treatment with Src inhibitor, PP2, also inhibited GPR56-mediated basal phosphorylation (Fig. S4*D*). Defactinib treatment leads to inhibition of p-Fak, as expected, and suppressed 10C7-induced phosphorylation of Pax (Fig. 6, *D* and *E*). No inhibition of Src phosphorylation was observed, and only a partial inhibition of baseline p-Pax levels was detected (Fig. 6, *D* and *E*). These data indicate that GPR56-mediated Src phosphorylation occurs upstream of Fak phosphorylation and that both Src and Fak can potentially phosphorylate Pax. Moreover, our findings suggest that GPR56-mediated Src–Fak signaling does not require activation of Gα_12/13,_ Gα_q,_ or RhoA.

## Discussion

**Figure 7:**
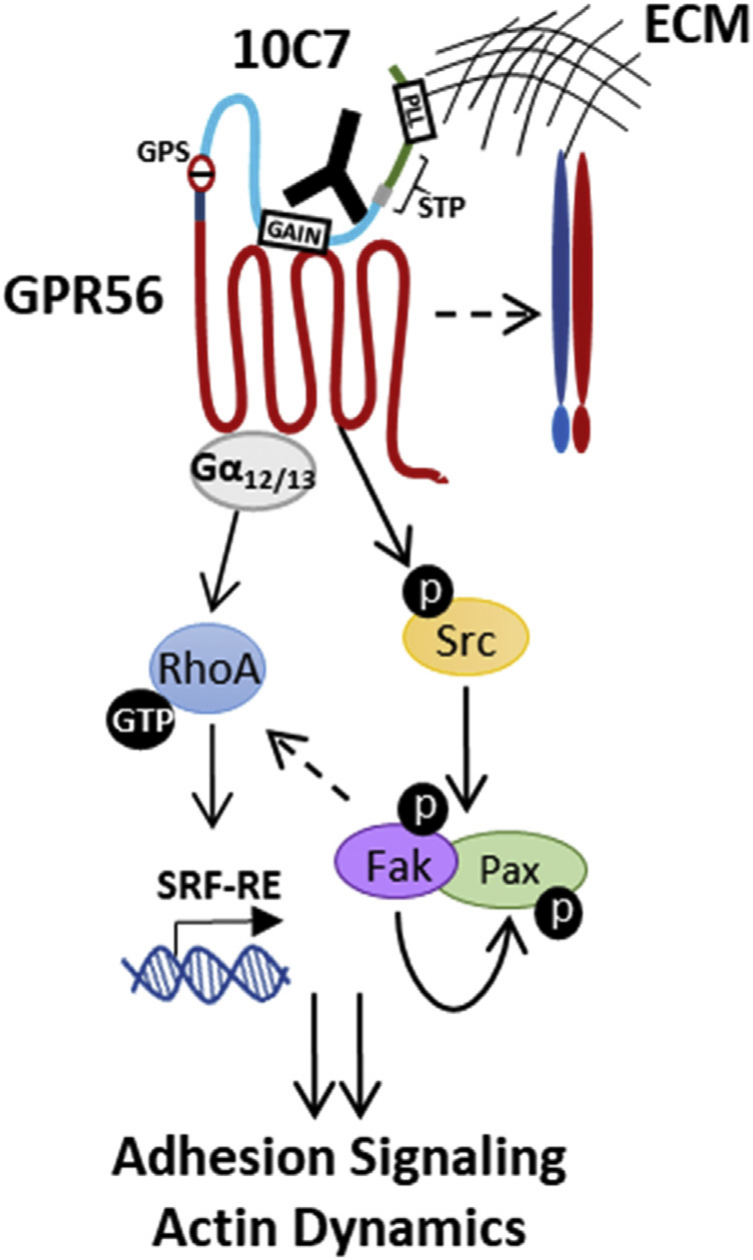
**Schematic of 10C7-induced GPR56 adhesion signaling model.** GPR56 activates G⍺_12/13_-RhoA–SRF signaling and promotes Src phosphorylation independent of RhoA. Src activation leads to phosphorylation of Fak and paxilllin. Binding of 10C7 to the GAIN domain of the ECD induces a conformational change of the ECD and/or promotes the potential interaction with other surface proteins (*e.g.*, integrins) to potentiate Src–Fak signaling and adhesion to the extracellular matrix (ECM). 10C7 potentiation of Src–Fak signaling enhances RhoA–SRF signaling downstream of G_12/13_ through an unknown mechanism. Deletion of the STP domain suppressed activation of Src–Fak signaling and inhibits 10C7 activity. Truncation of the PLL domain or inhibition of receptor autoproteolysis *via* mutation of the GPS site decreases constitutive RhoA–SRF signaling, yet is dispensable for 10C7-induced activation of RhoA–SRF or Src–Fak signaling. GPR56 likely coordinates activation of RhoA–SRF and Src–Fak signaling pathways to regulate adhesion and other cellular processes.

GPR56 has been shown to have essential functions in many physiological processes, and emerging evidence has established a critical role for GPR56 in tumor progression (2, 3, 4, 10, 12, 13, 14, 15). However, due to its intricate structure and lack of high-affinity receptor-specific ligand(s), the signaling mechanism of GPR56 still remains poorly understood. In this study, we generated a unique anti-GPR56 mAb, 10C7, directed against the ECD in order to interrogate the signaling mechanism(s) of recombinant and endogenous GPR56. Mapping of the mAb binding site using ECD deletion mutants showed that 10C7 binds within the GAIN domain. Based on our findings, we propose a model by which GPR56 can activate both G_12/13_-RhoA–SRF and Src–Fak signaling to regulate adhesion in normal and cancer cells (Fig. 7). Treatment with 10C7, or possibly an endogenous ligand, can potentiate Src phosphorylation leading to subsequent phosphorylation of Fak and Pax. 10C7 stimulation enhances RhoA–SRF activation in a Src-dependent manner. GPR56-mediated basal Src–Fak signaling and 10C7 potentiation of Src–Fak and RhoA–SRF signaling requires the STP region of the NTF, specifically the C-terminal portion spanning the PLL-GAIN linker and first two amino acids of the GAIN domain (a.a. 161–177), which may be involved in a conformational shift of the receptor. This conformational change may promote association of the NTF with the CTF stalk region, an extracellular loop of the TM, and/or potentially a coreceptor to transmit signaling. Interestingly, Jeong *et al.* (43) demonstrated synergistic activities of GPR56 and α3β1 integrin during cerebral cortical development, which may account for GPR56 activation of integrin partners such as Src–Fak. Additionally, GPR56-interacting proteins including CD81 and collagen III (24, 44) have also been shown to associate with integrins (45, 46, 47). The STP region overlaps with the PLL domain, which shares sequence similarity to LNS domains, which can mediate cell adhesion (19, 48). Deletion of residues within the STP and PLL regions has been shown to inhibit GPR56-mediated interactions with extracellular matrix proteins (7, 24, 35). This is consistent with our findings that both ΔPLL and ΔSTP mutant cell lines exhibited decreased adhesion to collagen. Furthermore, ΔPLL showed that lower basal RhoA-SRF signaling compared with WT and ΔSTP failed to activate Src–Fak phosphorylation, suggesting that N terminal of GPR56 (a.a. 26–177) plays an important role in regulating adhesion signaling. Furthermore, we showed that 10C7-induced Src–Fak signaling enhanced RhoA–SRF signaling through an unknown mechanism. Previously, Iguchi *et al.* (25) also showed that a polyclonal anti-GPR56 antibody could induce RhoA activation and SRE-mediated reporter activity. Several studies have demonstrated that Rho guanine nucleotide exchange factors (RhoGEFs) can bind and function as substrates for Fak (49, 50, 51). Fak activation of RhoGEFs could play a role in controlling active RhoA levels downstream of Src, thus leading to increased SRF signaling. The manner by which these GPR56-mediated signaling pathways function to coordinate the regulation of actin dynamics and cell adhesion requires further investigation.

Different stalk-dependent and -independent models have been proposed to provide structural and mechanistic insight into how extracellular interactions with GPR56 trigger intracellular signaling activity. However, it is possible that these models function in concert with each other or the GPR56 activation mechanism(s) may essentially be ligand- and/or pathway-dependent. We showed that 10C7 cointernalized with GPR56 (Fig. 1*G*), which initially suggested two potential scenarios of receptor activation; (1) 10C7 binds the NTF of the ECD and NTF dissociation is not required for potentiation of Src–Fak signaling, or (2) 10C7 directly binds the stalk region of the CTF and NTF dissociation may or may not occur. However, we showed that 10C7 binding occurs within the GAIN domain of the NTF (a.a. 177–382), suggesting the former scenario to be more accurate. In fact, 10C7 was shown to bind the GPS cleavage-deficient mutant, T383A, and potentiate both RhoA–SRF and Src–Fak signaling similar to WT. While we observed that overexpression of the T383A mutant exhibited a slight reduction in basal SRF activity compared with WT (approximately two- to three-fold), a previous report showed that mutation of T383A did not affect SRF signaling (20). This variation may be attributed to differences in T383A surface expression. Nonetheless, these findings suggest that NTF dissociation is not required for RhoA–SRF nor Src–Fak signaling. A similar GPR56 internalization pattern was shown for both 10C7 and a commercial mAb, which binds the myc-tag at the N terminal of recombinant GPR56, though the myc-tag mAb did not activate Src-Fak phosphorylation (Fig. 1*G* and Fig. S2, *B* and *C*). Furthermore, 10C7 cointernalized with ΔSTP, yet failed to potentiate signaling. These findings suggest that receptor internalization alone is not sufficient to promote GPR56 activation of Src–Fak signaling, yet whether 10C7 alters the route or rate of internalization requires further study. We theorize that 10C7 binding to the GAIN domain induces a conformational change involving the STP region of the ECD (within a.a. 161–177) that promotes its association with either the residues of the CTF, extracellular loop, and/or coreceptor, which may be obligate to activate Src–Fak signaling. Since ΔSTP was able to transduce basal constitutive RhoA–SRF signaling similar to WT, it suggests a model by which the N terminal of the PLL plays a more important role in the absence of exogenous agonist and independent of Src activation. Thus, GPR56 activation of RhoA–SRF and Src–Fak signaling appears to be stalk-independent. Consistent with the stalk-independent model, Ohta *et al.* (52) generated GPR56 mAbs and showed that agonistic mAbs could enhance interaction of NTF with the CTF. However, they utilized activation of G_q_ and inhibition of cell migration as a readout for agonist activity. Furthermore, the use of monobodies targeting different regions of the ECD supports stalk-independent regulation of GPR56-mediated SRE activity (23). Based on findings by Kishore *et al.*, (20) GAIN domain cleavage of the NTF was not necessary for GPR56-mediated basal activation of the SRF reporter, yet removal of the NTF did not affect signaling. Yet, truncation of the entire ECD including the CTF stalk region significantly abrogated SRF reporter activity (20). In our hands, myc-tagged mutants with deletion of the NTF (ΔNT, Fig. 1, *E*–*G* and Fig. S1*B*) or the entire ECD (not shown) failed to express at the cell surface. Thus, the role that the residues of stalk region play in GPR56-mediated RhoA–SRF and Src–Fak signaling remains to be further resolved.

Expression levels of Src and Fak are elevated in CRC and increased Src–Fak activation has been implicated in tumor growth and metastasis (37, 38, 39, 40, 53). Similarly, GPR56 is highly upregulated in CRC and shown to promote proliferation of CRC cells and tumor growth *in vivo*. Therefore, we utilized CRC cell lines to investigate endogenous effects of GPR56 on Src–Fak signaling. Similar to 293T cells, 10C7 potentiated Src–Fak phosphorylation in CRC cell lines and GPR56 knockdown suppressed Src–Fak signaling and cell adhesion (Fig. 5, *C*–*G*). Previous studies have demonstrated that loss of GPR56 in CRC cells leads to decreased migration, invasion, and resistance to chemotherapy (12, 13, 16); all of which may potentially be regulated through the Src–Fak pathway. In fact, therapeutic inhibitors of Src have been shown to suppress CRC cell growth and adhesion and sensitize cells to chemotherapy (54, 55, 56). Consistently, we showed that inhibition of Src suppressed 10C7 potentiation of adhesion in HT-29 cells (Fig. 5*H*). Moreover, both high GPR56 expression and activated Src–Fak have been associated with poor prognosis in CRC (12, 13, 53, 57). In melanoma, where GPR56 plays an inhibitory role, Millar *et al.* (58) showed by ICC that GPR56 depletion leads to increased phosphorylation of Fak. This suggests that GPR56 may have opposing roles in different cancers and may be dependent on differential expression of its natural ligands or cross talk with other signaling pathways. Based on these findings, it will be of interest to further explore the role of GPR56-mediated Src–Fak signaling in the regulation of tumor progression and drug resistance.

In conclusion, we generated a high-affinity anti-GPR56 mAb that can function as a biological tool to delineate the novel activation mechanism and signaling pathways of GPR56. Our findings uncover a unique ECD-dependent mechanism by which GPR56 potentially activates Src–Fak signaling to regulate normal and pathological processes. Therapeutic targeting of GPR56 to suppress Src–Fak and other associated signaling pathways could hold significant potential for the treatment of cancers with high expression of GPR56, particularly CRC.

## Experimental procedures

### Plasmids and cloning

Reporter vector pGL4.34[luc2P/SRF-RE/Hygro] was purchased from Promega. The myc-tagged GPR56 wild-type (GPR56 WT) vector (pIRESpuro3-myc-hGPR56) encoding amino acids 26 to 693 was previously described (13) and the mutant GPR56ΔSTP vector (pIRESpuro3-myc-hGPR56ΔSTP) lacking the STP domain was generated in a similar manner. Briefly, the sequence encoding GPR56ΔSTP (amino acids 26–693 with deletion of 108–177 within the ECD) was subcloned from BC-deltaSTP-GPR56 and fused with sequences encoding a Myc tag at the N terminus and cloned downstream of a sequence encoding the CD8 signal peptide (MALPVTALLLPLALLLHAA) in the vector pIRESpuro3 (Clontech). BC-deltaSTP-GPR56 was from Lei Xu (Addgene, 44198). For antibody generation, the sequence encoding the native signal peptide and ECD of GPR56 (amino acids 1–400) was subcloned from pCAG-hGPR56-IRES-GFP (from Christopher A Walsh, Addgene 52297) into pCEP4 (Invitrogen) and fused to a C-terminal 6x His-tag (GPR56ECD-6xHis).

### Commercial antibodies, chemical inhibitors, and other reagents

Commercial antibodies were used in accordance to manufacturer’s guidelines. For western blot: GPR56 (H00009289-B01P) from Abnova (Fig. 5*D*); pSrcY416 (6943), Src (2123), pFakY397 (8556), Fak (3285), p-paxillinY118 (2541), β-actin (3700), myc-tag (clone 9E11; 2276) from Cell Signaling; Gα_13_ (sc-293424), Gα_12_ (sc-515445), and Gα_q_ (sc-136181) from Santa Cruz Biotechnology; and paxillin (610863) from BD Biosciences. For ICC and cell-based binding assays: anti-myc-tag-Cy3 (clone 9E11; C6594) from Sigma, myc-tag (clone 9E11; 2276) from Cell Signaling, and secondary goat anti-mouse-Alexa-555 and goat anti-human-Alexa-555 (Life Technologies). The cell-permeable C3 transferase-based Rho inhibitor 1 was from Cytoskeleton. Saracatinib and defactinib were from Selleck Chemicals. hIgG1 isotype control was from Fisher Scientific.

### Cell culture, transfection, and stable cell line generation

HEK293T (293T), DLD-1, and HT-29 cells were purchased from ATCC. Cell lines were authenticated utilizing short tandem repeat profiling, routinely tested for *mycoplasma*. 293T cells were cultured in DMEM and colon cancer cell lines in RPMI medium and supplemented with 10% fetal bovine serum and penicillin/streptomycin. Transient transfections were performed in 6-well plates (1.5 μg DNA/well) using jetPRIME (Polypus Transfection). SMARTtpool siRNA control, Gα_12_ (L-008435-00-0005), Gα_13_ (M-009948-00-0005), and Gα_q_ (L-008562-00-0005) from Horizon Discovery were transfected at a final concentration of 50 nM. To generate bulk stable 293T cell lines, cells were transfected with GPR56 WT, ΔSTP, ΔPLL, ΔNT, or T383A or control vector and selected in 1 μg/ml puromycin. DLD-1 and HT-29 shRNA control (pLKO.1, shCTL) and GPR56 (shGPR56) knockdown cell lines were generated by lentiviral infection as previously reported (13).

### Generation of anti-GPR56 monoclonal antibody

The anti-GPR56 mAb, 10C7, was generated together with ProMab Biotechnologies (Richmond, CA, USA, promab.com). Briefly, GPR56ECD-6xHis was purified using a Ni-NTA column and mice were immunized with the purified ECD. After a series of immunization and cloning steps, hybridoma clones were selected and the supernatants screened for binding specificity by ELISA and ICC. The mouse variable heavy and light chain (V_H_ and V_L_) sequences were amplified by PCR, cloned, and then sequenced. To produce a mouse–human chimera 10C7 mAb, the mouse V_H_ and V_L_ were subcloned into pCEP4 expression vectors containing the constant region of human IgG1 heavy chain (C_H_) and kappa light chain (C_L_) _as_ previously described (59). Large-scale mAb production and purification were performed as reported earlier (31).

### Western blot analysis

For western blots, protein extraction was performed using RIPA buffer (Sigma) supplemented with protease/phosphatase inhibitors. Cell lysates were incubated at 37 °C for 1 h and diluted in laemmli sample buffer prior to loading on SDS-PAGE. HRP-labeled secondary antibodies were utilized for detection with the standard ECL protocol. Western blots shown are representative of at least three independent experiments. Quantification was normalized to vector or vehicle-treated cells and performed using ImageJ.

### Immunocytochemistry and cell-based binding assays

For ICC, HEK293T or DLD-1 cells were seeded into 8-well chamber slides and incubated overnight. The next day, cells were treated with 10C7, myc-tag-Cy3 mAb, or hIgG1 isotype control at 37 °C for 1 h to allow binding and internalization of mAbs. Cells were washed with PBS, fixed in 4% formalin, permeabilized in 0.1% saponin, then incubated with goat anti-human-Alexa-555 for 1 h at room temperature. Nuclei were counterstained with TO-PRO-3. Images were acquired using confocal Leica TCS SP5 microscope with LAS AF Lite software. Quantification of 10C7 binding was performed for approximately 30 to 40 cells from multiple images using ImageJ. Whole-cell-based binding assays were performed as previously described (59). Briefly, cells were seeded onto poly-d-lysine-coated 96-well plates and incubated overnight. Serial dilutions of 10C7, myc-tag mAb, of hIgG1 were added for 2 h at 4 °C. Plates were washed in PBS, fixed (with or without permeabilization as indicated), incubated with Alexa-555 labeled secondaries for 1 h at room temperature, and washed. Fluorescence intensity was quantified using a Tecan Infinite M1000 plate reader and fluorescent signals were normalized to 0 at baseline level and 1.0 at maximum level fluorescence for each cell line.

### Luciferase reporter assays

HEK293T cells transiently cotransfected with pGL4.34 (SRF-RE) and other expression vectors, as indicated, and were plated at 2500 cells/well in 96 half-well plates. Serial dilutions of 10C7 and/or chemical inhibitors were added and allowed to incubate at 37 °C overnight. Luciferase activity was measured using Pierce Firefly Luciferase Glow Assay Kit according to manufacturer’s protocol using an EnVision mulitlabel plate reader (PerkinElmer). AlamarBlue (ThermoFisher) was used for normalization to cell viability and fluorescence quantified at 530/590 nm using Tecan Infinite M1000 plate reader. Each condition was performed in triplicate, n ≥ 3.

### Adhesion assays

Cell were seeded in a 96-well collagen I-coated plate at 15,000 cells/well (1.5 × 10^5^ cells/ml), in the appropriate culture medium and incubated at 37 °C for the indicated time points. For antibody treatment studies, 293T and colorectal cancer cells were treated with 3 μg/ml (or 20 nM) and 20 μg/ml (or 130 nM) antibody, respectively, for 1 h prior to cell seeding. To determine the effect of Src inhibition on 10C7-induced adhesion, HT-29 cells were pretreated with saracatinib (10 μM) or DMSO vehicle for 1 h before addition of antibodies. Nonattached cells were washed with PBS, fixed with 4% formalin for 15 min, and labeled with 5 mg/ml crystal violet for 10 min. Plates were washed with PBS to remove unbound dye. Crystal violet from adherent cells was solubilized using 2% SDS, and absorbance was measured at 595 nm using Tecan Infinite M1000 plate reader.

### Statistical analysis

Statistical analysis was performed using GraphPad Prism 5 software. For all experiments n ≥ 3 and data are presented as mean ± standard error of the mean (S.E.) or standard deviation (SD) for bar graphs. Kd and EC_50_ values were determined using logistic nonlinear regression models. For quantification of ICC, significance was determined using Student’s *t*-test. For adhesion assays, differences between groups were analyzed by two-way ANOVA. Other multiple comparisons used one-way ANOVA and Tukey’s post-hoc analysis. *p* ≤ 0.05 was considered statistically significant.

## Data availability

All data are contained within the article.

## Conflict of interest

The authors declare that they have no conflicts of interest with the contents of this article.
