# Supplementary material for: Anti-GPR56 monoclonal antibody potentiates GPR56-mediated Src-Fak signaling to modulate cell adhesion
Source: J Biol Chem. 2021 Jan 8;296:100261. doi: 10.1016/j.jbc.2021.100261 (PMC7948743; doi:10.1016/j.jbc.2021.100261)
Supplement: Supplemental Figures S1–S4 [file mmc1.pdf]

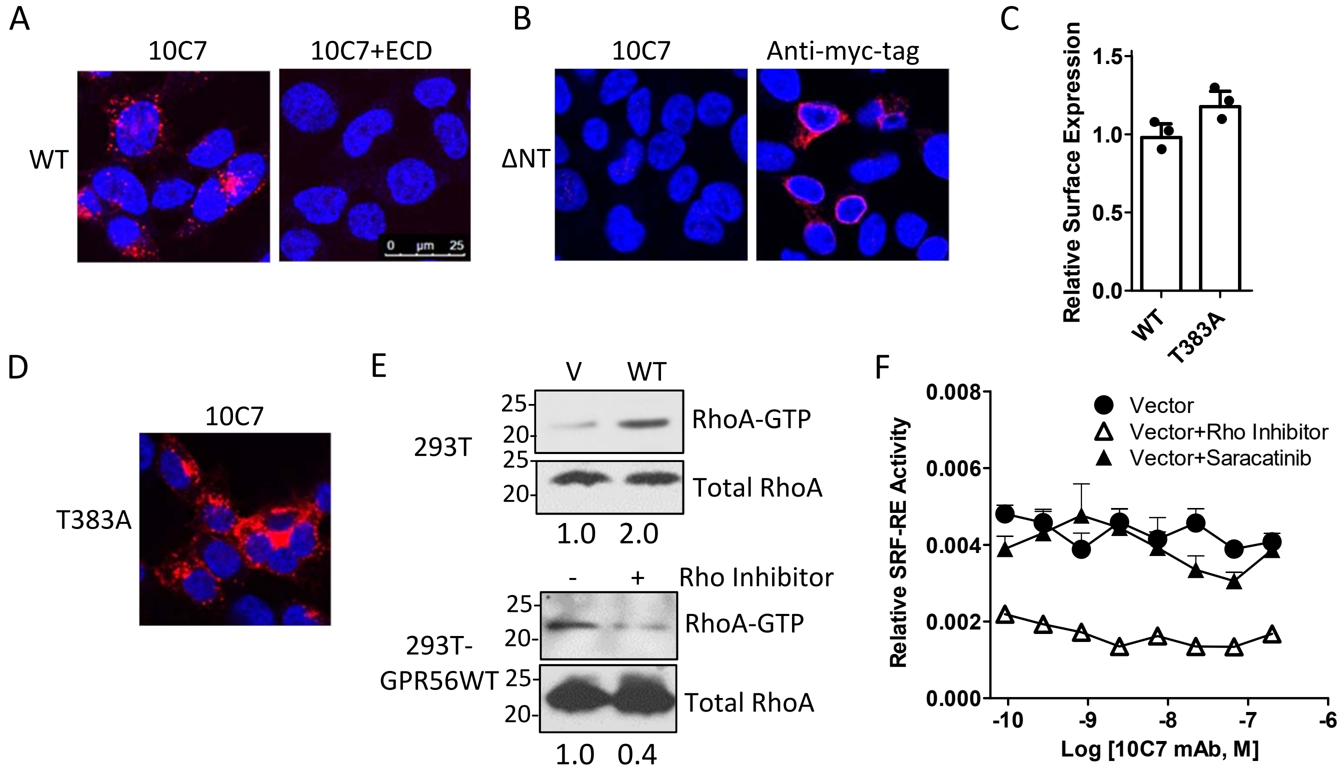

Supplementary Figure S1. 10C7 binding to GPR56 mutants and inhibitor effects on RhoA-SRF signaling. A Confocal microscopy images of purified GPR56 ECD inhibition of 10C7 binding and internalization. 10C7 was pre-incubated +/- purified ECD for 30 min at room temp., the mixture was then added to 293T-GPR56 WT cells for 1 hr at 37ºC. 10C7 binding was detected using Alexa-555 labeled anti-human secondary Ab. B, 293T-ΔNT cells were fixed, permeabilized and then incubated with 10C7 (followed by Alexa-555 labeled anti-human Ab) or anti-myc-tag-Cy3 for 1hr at room temperature. C, Quantification of relative surface expression of WT verses T383A based on myc-tag mAb binding to live cells at 4ºC. Error bars, S.D. D, 10C7 binding and internalization after 1hr at 37ºC in cells with stable overexpression of GPR56-T383A mutant. E, RhoA activation assay shows increased levels in GPR56WT compared to vector cells. Rho Inhibitor I effectively inhibits active RhoA levels in 293T-GPR56 cells. Cells were starved, pre-treated with +/- Rho inhibitor I for 3 hrs, then serum stimulated for 15 min. RhoA-GTP pulldown assay (Cytoskeleton, #BK036) that employs the Rho binding domain of the Rho effector, Rhotekin, was performed according to manufacturer's protocol. F, Effects of Rho inhibitor I and Src inhibitor (saracatinib) on SRF-RE response in vector cells. Error bars are S.E. (n=3).

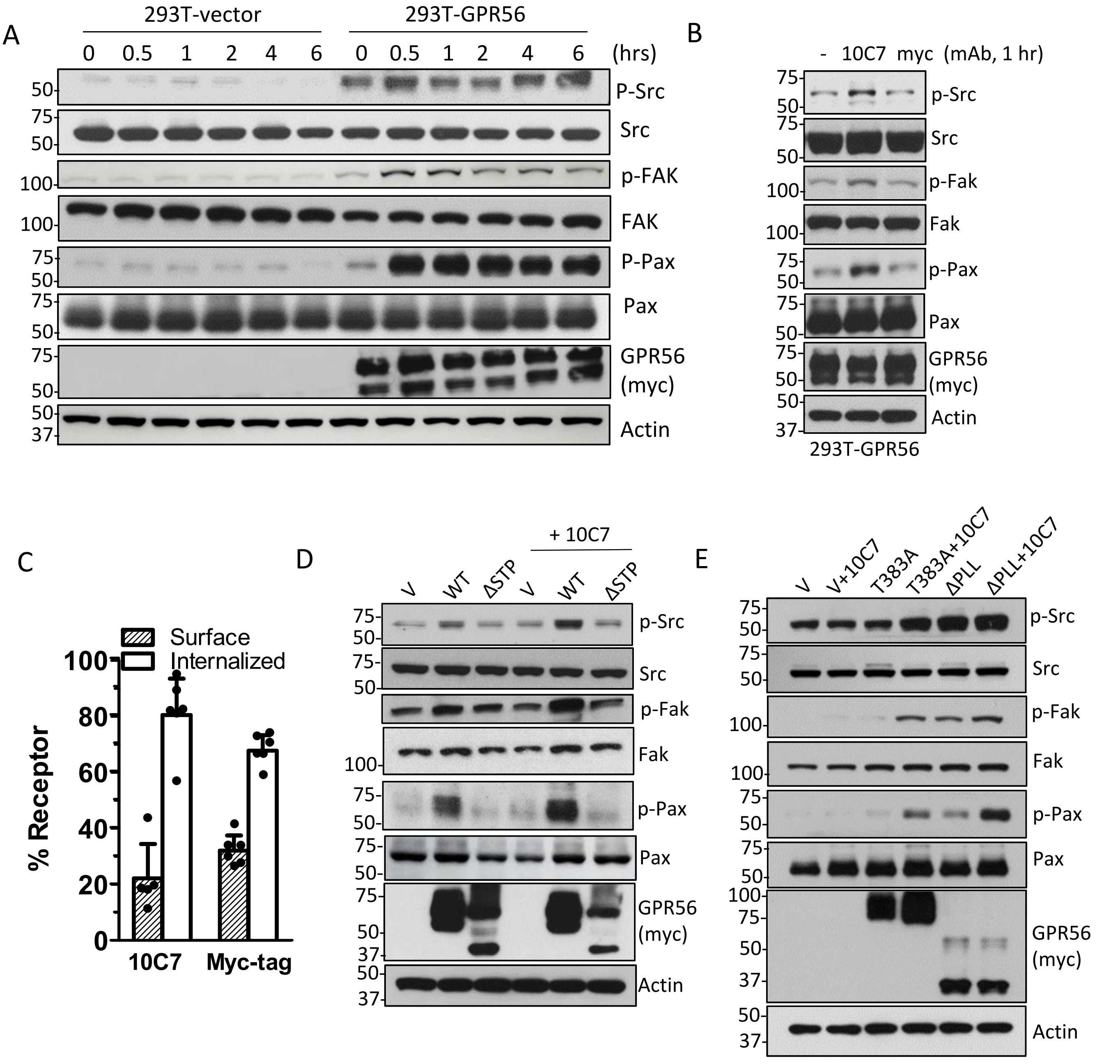

Supplementary Figure S2. 10C7 effects on Src-Fak signaling. A, Western blot of time-dependent effects of 10C7 treatment on Src/Fak signaling in 293T vector and GPR56 stable cells. B, 10C7 and not anti-myc-tag mAb (myc) potentiates Src-Fak signaling in stable 293T-GPR56 cells. C, Quantification surface and internalized GPR56 using fluorescence cell-based binding assay. GPR56 stable cells were treated with indicated mAbs for 1 hour, then fixed. Total and surface mAb-GPR56 complexes were quantified from Alexa-555 labeled secondary Ab binding to permeabilized and non-permeabilized cells, respectively. Percent internalized receptor was estimated by (Total-Surface/Total x 100%). Error bar, S.D. D, Western blot showing transient transfection of GPR56, but not ΔSTP, in 293T cells enhances phosphorylation of Src, Fak, and paxillin which is further augmented by 10C7 treatment. E, Western blot showing 10C7 increases Src/Fak signaling in 293T cells transiently transfected with T383A and ΔPLL mutants. D, All treatments were performed with 3 μg/ml (or 20 nM) 10C7 or anti-myc mAb for 1 hour unless otherwise indicated.

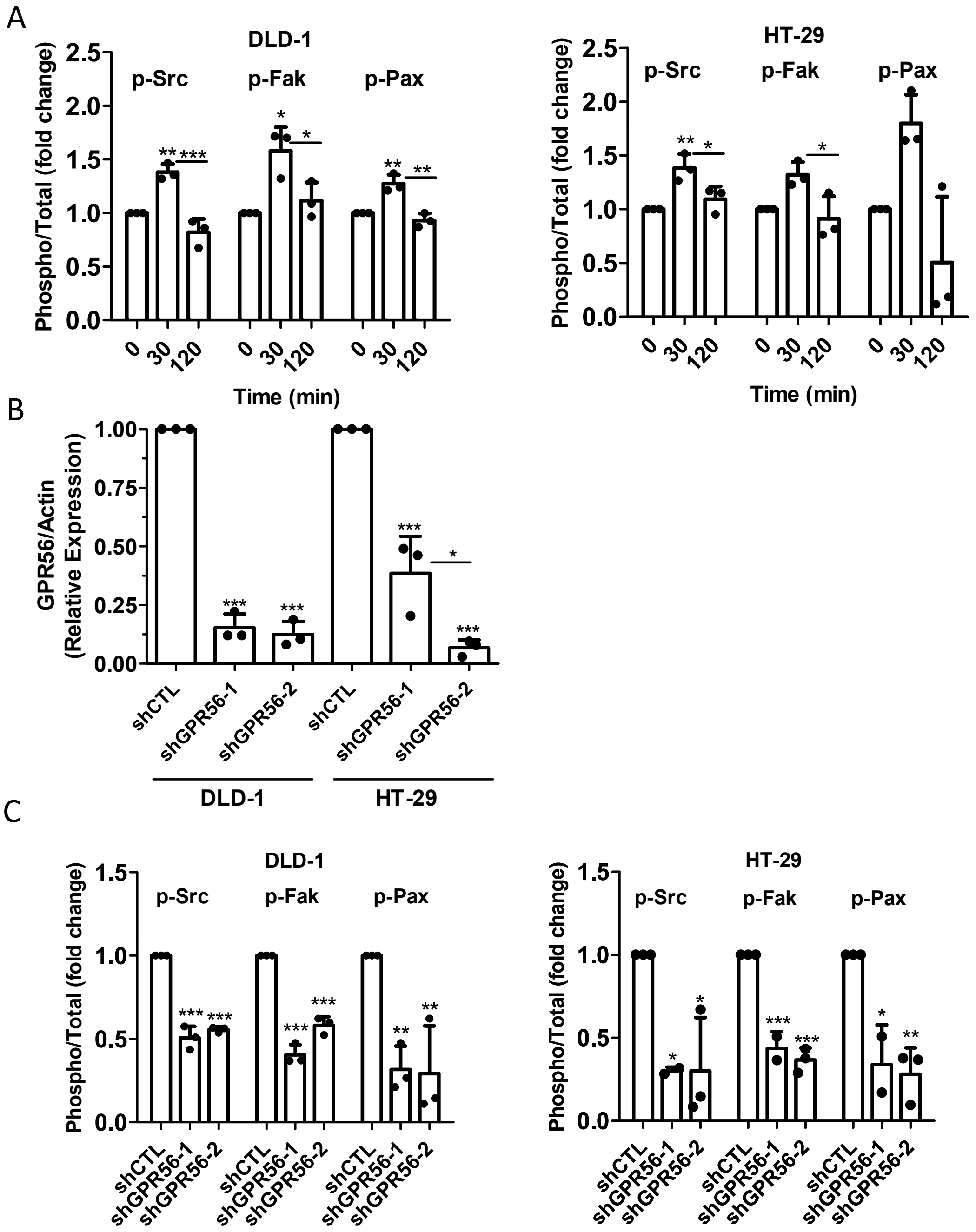

Supplementary Figure S3. Quantification of western blots of colorectal cancer cell lines in Figure 5. A. Quantification of p-Src, p-Fak, and p-Pax in colon cancer cell lines with 10C7 treatment as shown in Fig. 5C. B, Quantification of GPR56 knockdown by shRNA based on protein expression as shown by western blot in Fig 5D. C, Quantification of changes in p-Src, p-Fak, and p-Pax in colorectal cancer cell lines with GPR56 KD as shown in Fig. 5C. All data represent at least three independent experiments. Statistical analysis performed by one-way ANOVA, * $p<0.05$, ** $p<0.01$, and *** $p<0.001$. Error bars, S.D.

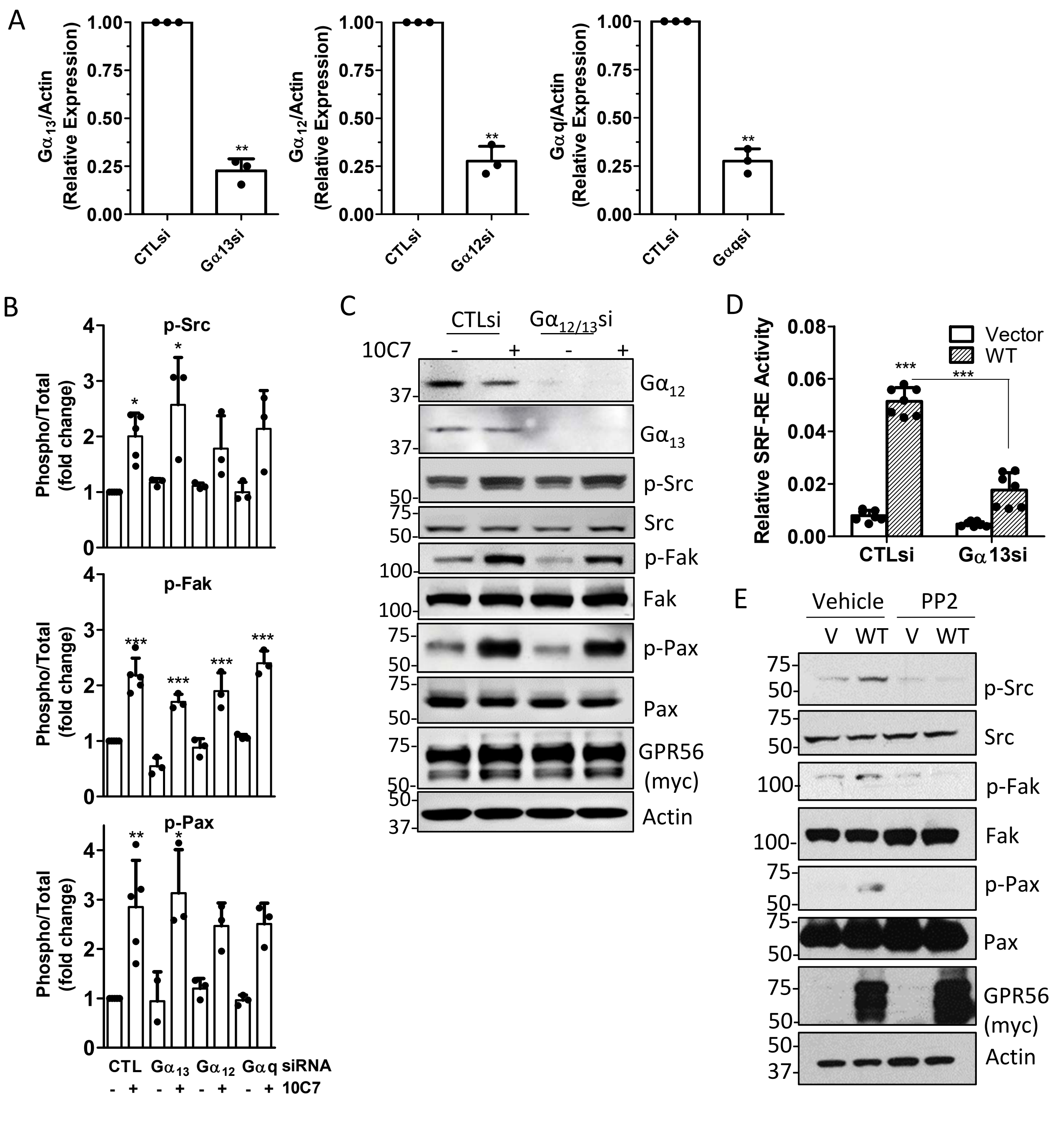

Supplementary Figure S4. GPR56-mediated activation of the Src-Fak pathway is not mediated by $G\alpha_{12/13}$ and is inhibited by PP2. A. Quantification of siRNA knockdown of $G\alpha_{12}$, $G\alpha_{13}$, and $G\alpha_q$ based on protein expression as shown by western blots in Figs 6A-B. B, Quantification of Src-Fak signaling based on at least 3 independent experiments as shown in Figs. 6A-B. C, Western blot showing double knockdown of $G\alpha_{12}$ and $G\alpha_{13}$ does not effect GPR56-mediated Src/Fak signaling. D, Effects of $G\alpha_{13}$ siRNA knockdown on basal SRF-RE response in 293T cells transiently transfected with vector or GPR56. E, Western blot shows 30 min treatment with 10μM Src inhibitor, PP2, suppresses GPR56 phosphorylation of Src, Fak, and paxillin. Statistical analysis performed by one-way ANOVA, * $p<0.05$, ** $p<0.01$, and *** $p<0.001$. Error bars, S.D.
